# The lactate clearance calculated using serum lactate level 6 h after is an important prognostic predictor after extracorporeal cardiopulmonary resuscitation: a single-center retrospective observational study

**DOI:** 10.1186/s40560-018-0302-z

**Published:** 2018-06-01

**Authors:** Takashi Mizutani, Norio Umemoto, Toshio Taniguchi, Hideki Ishii, Yuri Hiramatsu, Koji Arata, Horagaito Takuya, Sho Inoue, Tsuyoshi Sugiura, Toru Asai, Michiharu Yamada, Toyoaki Murohara, Kiyokazu Shimizu

**Affiliations:** 1 Cardiovascular Center, Ichinomiya Municipal Hospital, Ichinomiya, Japan; 2 Department of Emergency, Ichinomiya Municipal Hospital, Ichinomiya, Japan; 3 Department of Cardiology, Nagoya University Graduate School of Medicine, Nagoya, Japan; 4 Department of Medical Engineering, Ichinomiya Municipal Hospital, Ichinomiya, Japan; 5 Department of Cardiology, Ichinomiya Municipal Hospital, 2-2-22 Bunkyo, Ichinomiya City, Aichi 491-8558 Japan

**Keywords:** Lactate clearance, Extracorporeal cardiopulmonary resuscitation, Cardiac arrest, In-hospital mortality

## Abstract

**Background:**

Serum lactate level can predict clinical outcomes in some critical cases. In the clinical setting, we noted that patients undergoing extracorporeal cardiopulmonary resuscitation (ECPR) and with poor serum lactate improvement often do not recover from cardiopulmonary arrest. Therefore, we investigated the association between lactate clearance and in-hospital mortality in cardiac arrest patients undergoing ECPR.

**Methods:**

Serum lactate levels were measured on admission and every hour after starting ECPR. Lactate clearance [(lactate at first measurement − lactate 6 h after)/lactate at first measurement × 100] was calculated 6 h after first serum lactate measurement. All patients who underwent ECPR were registered retrospectively using opt-out in our outpatient’s segment.

**Result:**

In this retrospective study, 64 cases were evaluated, and they were classified into two groups according to lactate clearance: high-clearance group, > 65%; low-clearance group, ≤ 65%. Surviving discharge rate of high-clearance group (12 cases, 63%) is significantly higher than that of low-clearance group (11 cases, 24%) (*p* < 0.01). Considering other confounders, lactate clearance was an independent predictor for in-hospital mortality (odds ratio, 7.10; 95% confidence interval, 1.71–29.5; *p* < 0.01). Both net reclassification improvement (0.64, *p* < 0.01) and integrated reclassification improvement (0.12, *p* < 0.01) show that adding lactate clearance on established risk factors improved the predictability of in-hospital mortality.

**Conclusion:**

In our study, lactate clearance calculated through arterial blood gas analysis 6 h after ECPR was one of the most important predictors of in-hospital mortality in patients treated with ECPR after cardiac arrest.

## Background

Extracorporeal cardiopulmonary resuscitation (ECPR) is one of the most powerful therapies after cardiopulmonary arrest (CPA) [1]. A prior study showed that earlier return of circulation leads to improvement of 30-day survival, surviving discharge rate, and clinical performance category (CPC) [2]. Because ECPR restarts systemic circulation forcibly, it is a very strong strategy when cardiopulmonary resuscitation or defibrillation is not effective. However, the guideline of the American Heart Association (AHA) cited insufficient evidence and limited the indication for ECPR [3].

According to AHA guidelines, ECPR may be considered for selected cardiopulmonary arrest patients in whom the suspected etiology of cardiac arrest is potentially reversible during a limited period of mechanical cardiorespiratory support [3]. Because the CPC or surviving discharge rate is very low after CPA or ECPR, the adequacy of continuation of ECPR should be considered [4]. Nevertheless, there have been very limited data of ECPR prognosis or risk factors [3]. Risk factors such as initial rhythm (shockable rhythm or non-shockable rhythm), old age, CPA without bystander, without bystander CPR, longer CPR duration time, and without defibrillation are well-known independent prognostic factors.

A prior study showed that early goal-directed hemodynamic optimization therapy is effective in cardiac arrest [5, 6]. Meanwhile, earlier improvement in lactate clearance (LC) was reported to lead to better prognosis in the treatment of sepsis [7, 8]. In addition, serum lactate-guided intensive care reduces hospital mortality in the treatment of sepsis [9]. Post-cardiac arrest syndrome (PCAS) is reported to be one of the sepsis-like syndromes [10]. Of course, lactate reduction is one of most important predictors of survival and neurological outcome after cardiac arrest [11]. Thus, we hypothesized that LC might be an indicator of ECPR effectiveness.

We investigated the association between LC and surviving discharge in cardiac arrest patients treated with ECPR.

## Methods

In this single-center retrospective observational study, we collected data on 98 patients treated with percutaneous cardiopulmonary support (PCPS) at our hospital between 2011 and 2016.

**Fig. 1:**
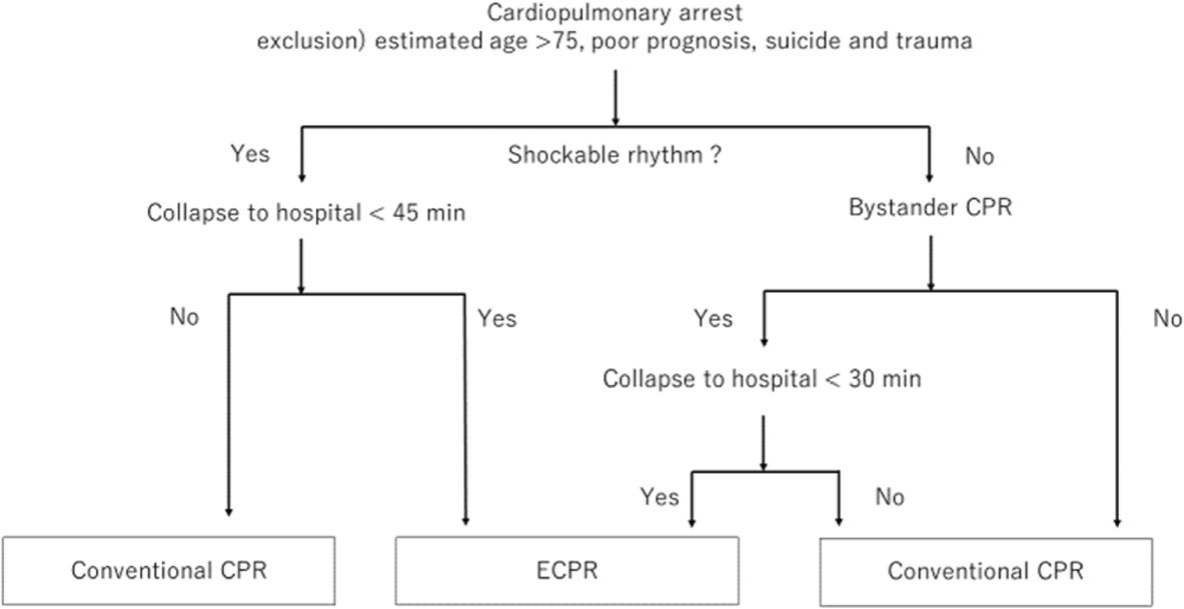
Decision tree of ECPR or conventional CPR with inclusion and exclusion criteria for ECPR

Among the 98 patients treated with PCPS at our hospital, 24 received PCPS before cardiac arrest; these cases were not defined as cases of ECPR and were excluded. Further, we also excluded patients with an etiology of aortic dissection. Finally, 4 patients who died before 6 h after ECPR and one patient whose serum lactate lose at 6 h were also excluded. Thus, in total, we evaluated 64 patients undergoing ECPR after cardiac arrest (Fig. 1). Their data, including their serum lactate levels and clinical courses, including that after ECPR, were retrospectively collected.

**Fig. 2:**
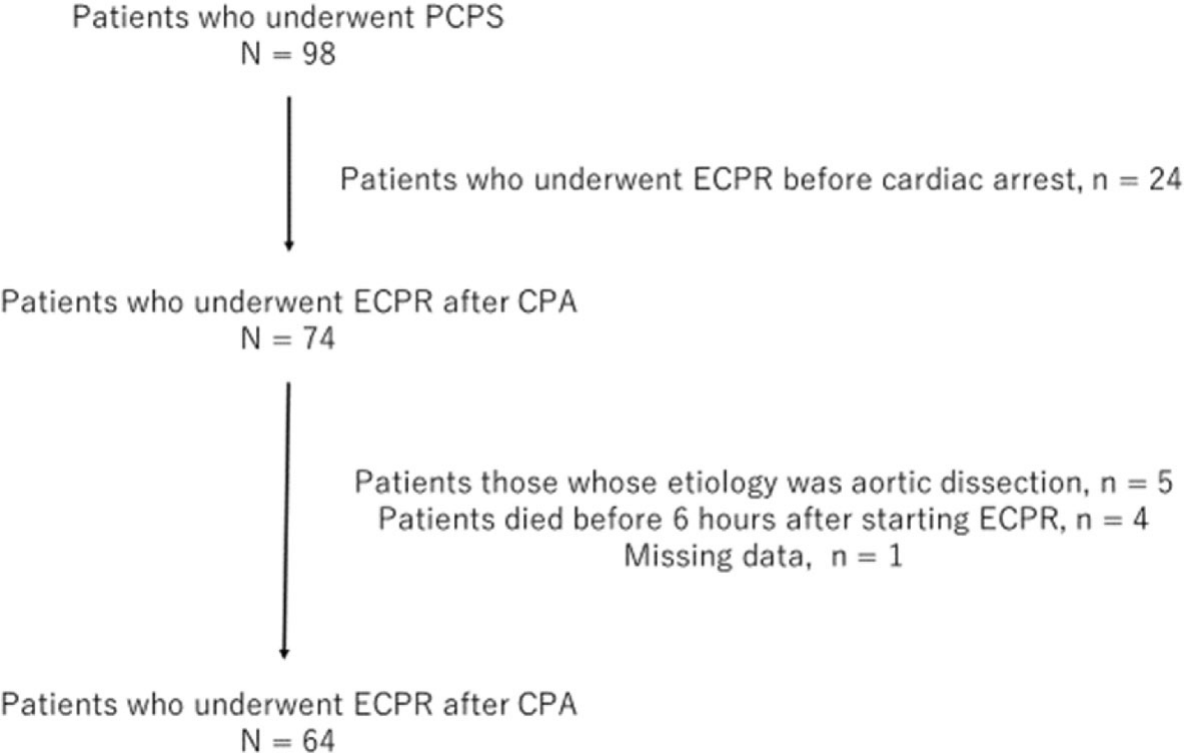
Design of this observation study

The exclusion criteria for ECPR were as follows: patients with estimated age > 75 years, those with no long-term prognosis, and those with dementia. Our in/exclusion flow chart is shown in Fig. 2. In addition, ECPR was not performed even if the cardiovascular physician decided it would not be effective for the patient.

After commencement of ECPR, we usually check the patients’ consciousness levels before leaving our catheter laboratory. In cases in which the Glasgow Coma Scale motor score was below 6, targeted temperature management (TTM) was initiated. Our TTM protocol is as follows: 34 °C for 48 h and a recovery temperature of 36 °C during the next 24 h.

The primary outcome was survival discharge, and the secondary outcomes were 30-day mortality and neurological outcomes.

### Serum lactate measurement and LC calculation

Arterial blood gas samples were immediately obtained from the arteries of all CPA patients in the emergency department of our hospital. The artery blood samples were immediately transferred to our laboratory and measured using RapidLab (Siemens AG, Erfurt, Germany). In case of ECPR, arterial blood gas analysis was performed every hour until the end of ECPR. Serum lactate was measured simultaneously with artery blood gas analysis. Blood sample was transferred to the laboratory and measured as soon as possible every time.

We calculated LC using the serum lactate level at the emergency department and at 6 h after admission using the following formula:

$$ \mathrm{LC}=\frac{\left(\mathrm{lactate}\ \mathrm{at}\ \mathrm{first}\ \mathrm{measurement}-\mathrm{lactate}\ 6\ \mathrm{hours}\ \mathrm{after}\right)}{\mathrm{lactate}\ \mathrm{at}\ \mathrm{first}\ \mathrm{measurement}}\times 100. $$

Patients with LC > 65% were included in the high-clearance group, and those with LC ≤ 65% were included in the low-clearance group.

**Fig. 3:**
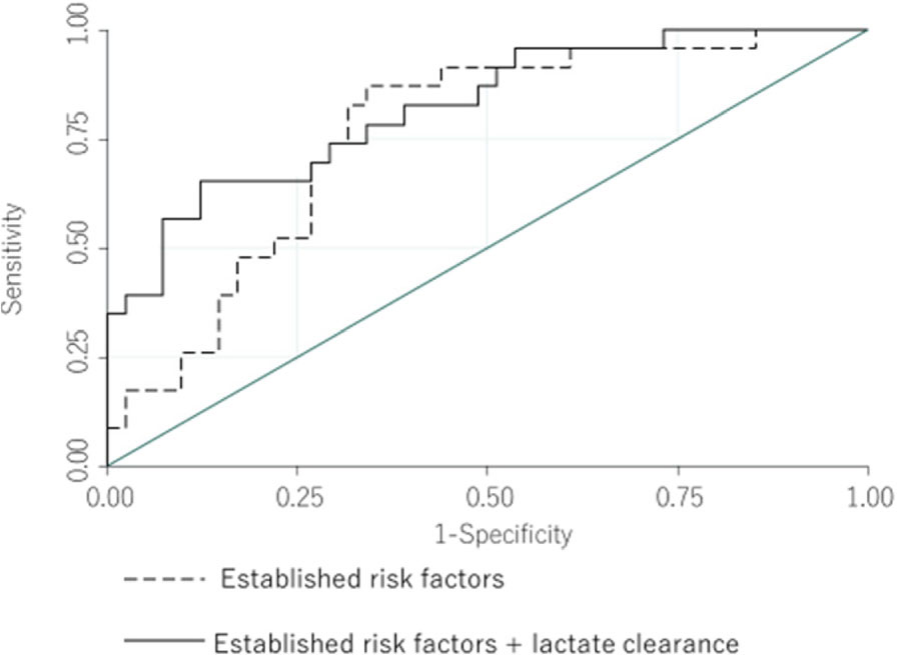
ROC comparison between established risk factors and + LC

We determined the cutoff value retrospectively. Receiver operating characteristic curve (ROC) analysis yielded a cutoff value of LC = 69% (sensitivity 0.48, 1 − specificity 0.43, AUC 0.75) (Fig. 3). In addition, we observed that the serum lactate level improved on early lactate-guided therapy [9]. The reported improvements in the lactate levels were 4.7–1.7 (64%) in the control group and 4.6–1.6 (65%) in the targeted group. Through consensus with ECPR specialists and analysts, we concluded that LC = 65% is a better cutoff value for ECPR.

### Statistical analysis

Statistical analysis was performed using JMP version 13.0 software (SAS Institute, Cary, NC, USA). Continuous variables were presented as median values with interquartile ranges according to the results of the normality test. Mann-Whitney *U* tests were conducted for comparison of continuous variables. Categorical variables were presented as frequencies and percentages and compared using chi-squared or Fisher exact tests. Intergroup differences in the continuous and categorical variables were evaluated using the Student *t* test and chi-squared test, respectively. Intergroup differences in mortality were evaluated using a logistic regression model. The significant variables from univariate analysis and the established risk factors were included in multivariate logistic regression analysis. We included the established risk factors (age, sex, initial rhythm, in-/out-hospital CPA, pH, and CRP duration) as confounders during multivariate logistic regression analysis. In addition, we calculated the area under the ROC (AUC), net reclassification improvement (NRI), and integrated discrimination improvement (IDI) to assess the accuracy of LC as a predictor when added to a baseline model with established risk factors. NRI indicates the relative number of patients with improved predicted probabilities for LC, whereas IDI represents the average improvement in predicted probabilities for LC after adding LC variables into the baseline model. In all analyses, *p* < 0.05 was considered statistically significant.

## Results

Patient characteristics are shown in Table 1. There were significant differences in total protein (low-clearance group: median, 4.9 g/dL; range, 4.0–5.6 g/dL; high-clearance group: median, 5.7 g/dL; range, 5.1–6.4 g/dL; *p* = 0.02), serum albumin (low-clearance group: median, 2.5 g/dL; range, 2.0–3.2 g/dL; high-clearance group: median, 3.3 g/dL; range, 2.6–3.8 g/dL, *p* = 0.01), and platelet (low-clearance group: median, 167,000; range, 148,000–209,000; high-clearance group: median, 213,000; range, 143,000–253,000 g/dL; *p* = 0.02). Except for total protein, serum albumin, and platelet, the baseline characteristics in both groups were well-matched.

**Table 1:** Patients’ background

	All cases *n* = 64	Low-clearance group *n* = 45	High-clearance group *n* = 19	*p* value
Age	70.8 (58.5–77.9)	71.7 (63.8–77.3)	64.1 (50.6–78.5)	0.30
Female (%)	17 (27)	14 (31)	3 (18)	0.35
Height cm	165 (157–172)	164 (156–170)	164 (156–170)	0.08
Weight kg	63 (54–72)	61 (53–72)	65 (55–75)	0.45
BMI	23 (21–25)	23 (21–25)	23 (21–26)	0.88
Diabetes (%)	23 (36)	17 (38)	6 (32)	0.78
Hypertension (%)	34 (53)	25 (56)	9 (47)	0.59
Dyslipidemia (%)	20 (31)	12 (27)	8 (42)	0.25
Current smoke (%)	19 (30)	10 (22)	9 (47)	0.07
Hemodialysis (%)	4 (6.3)	2 (4.4)	2 (11)	0.58
Prior PCI (%)	13 (20)	10 (22)	3 (16)	0.56
Prior CABG (%)	6 (9.4)	4 (8.9)	2 (11)	1.00
OMI (%)	15 (23)	10 (22)	5 (26)	0.75
Initial rhythm				0.48
VF/pulseless VT (%)	38 (59)	25 (56)	13 (68)	
PEA/asystole (%)	26 (41)	20 (44)	6 (32)	
Location				0.42
In-hospital (%)	45 (55)	23 (51)	12 (63)	
Out-hospital (%)	29 (45)	22 (49)	7 (37)	
CPR duration (min)	24 (12–45)	24 (16–46)	25 (10–46)	0.67
Laboratory data				
pH	7.03 (6.92–7.15)	7.03 (6.89–7.14)	7.09 (6.92–7.20)	0.54
Lactate mmol/L	11.8 (9.9–14.8)	11.7 (9.7–14.9)	12.8 (10.1–14.2)	0.74
Total protein g/dL	5.2 (4.3–6.1)	4.9 (4.0–5.6)	5.7 (5.1–6.5)	0.02
Albumin g/dL	2.9 (2.1–3.5)	2.5 (2.0–3.2)	3.3 (2.6–3.8)	0.01
BUN mg/dL	19 (16–28)	20 (17–27)	19 (14–31)	0.59
Creatinine mg/dL	1.1 (1.0–1.5)	1.1 (0.9–1.4)	1.1 (1.0–1.5)	0.74
Total cholesterol mg/dL	129 (80–164)	98 (70–164)	142 (97–178)	0.15
Low-density lipoprotein mg/dL	70 (55–109)	62 (54–105)	95 (70–127)	0.11
High-density lipoprotein mg/dL	28 (20–35)	25 (18–33)	28 (24–40)	0.16
Triglyceride mg/dL	71 (42–96)	58 (32–103)	84 (71–94)	0.26
Hemoglobin g/dL	11.6 (9.5–13.8)	11.0 (8.3–13.9)	12.2 (11.2–13.6)	0.14
White blood cell count 10^3^/μL	124 (97–168)	121 (96–16)	137 (106–194	0.21
Platelet 10^4^/μL	16.7 (11.9–21.6)	14.8 (9.8–20.9)	21.3 (14.3–25.3)	0.02
C-reaction protein mg/dL	0.19 (0.06–3.13)	0.19 (0.07–2.56)	0.16 (0.05–4.83)	0.91

### Diagnosis and follow-up data

The final diagnosis is shown in Table 2. In the first 30 days, the survival rate was significantly higher in the high-clearance group (12 cases, 63%) than in the low-clearance group (12 cases, 27%; *p* ≤ 0.01). The survival discharge rate was significantly higher in the high-clearance group (12 cases, 63%) than in the low-clearance group (11 cases, 24%; *p* < 0.01) (Table 3). Neurological outcome at discharge was better in the high-clearance group than in the low-clearance group (Table 3).

**Table 2:** Final diagnosis

Diagnosis	All cases *n* = 69	Low-clearance group *n* = 45	High-clearance group *n* = 19	*p* value
				0.48
Cardiac rapture (%)	11 (15.9)	6 (13)	1 (5.3)	
Electrical storm (%)	6 (8.7)	3 (6.7)	3 (16)	
Heart failure (%)	4 (5.8)	5 (11)	2 (11)	
Ischemic heart disease (%)	32 (46.4)	21 (47)	10 (53)	
PE (%)	3 (4.4)	2 (4.4)	1 (5.3)	
Myocarditis (%)	2 (2.9)	1 (2.2)	1 (5.3)	
Other	11 (15.9)	7 (16)	1 (5.3)

**Table 3:** Primary outcome and secondary outcome

	All cases *n* = 64	Low-clearance group *n* = 45	High-clearance group *n* = 19	*p* value
Surviving discharge	23 (36)	11 (24)	12 (63)	< 0.01
30-day survival (%)	24 (38)	12 (27)	12 (63)	< 0.01
Neurological outcome (CPC)				< 0.01
1 (%)	16 (25)	8 (18)	11 (58)	
2 (%)	3 (4.7)	2 (4.4)	1 (5.3)	
3 (%)	1 (1.6)	1 (2.2)	0 (0.0)	
4 (%)	1 (1.6)	1 (2.2)	0 (0.0)	
5 (%)	43 (67)	34 (76)	7 (37)	

In the univariable and multivariable logistic regression analyses for surviving discharge, LC was an independent predictor for surviving discharge (odds ratio, 7.10; 95% confidence interval, 1.71–29.5; *p* < 0.01). CPR duration time, location (in-/out-hospital CPR), and pH were also successful independent predictors for surviving discharge (Table 4). The NRI and IDI are shown in Table 5. Adding LC to the established risk factors improved predictability of surviving discharge after ECPR.

**Table 4:** Multi-logistic analysis for surviving discharge

	Univariable			Multivariable		
	Odds ratio	Confidential interval	*p* value	Odds ratio	Confidential interval	*p* value
Age	0.98	0.95–1.03	0.63	1.02	0.96–1.10	0.49
Initial rhythm (VF and pulseless VT/asystole and PEA)	1.47	0.51–4.22	0.46	1.42	0.32–6.27	0.64
Location(out/in)	1.17	0.42–3.26	0.64	10.0	1.35–75.0	0.01
CPR duration	1.02	0.99–1.05	0.22	1.08	1.01–1.12	0.02
pH	25.2	1.24–509	0.03	247	4.16–15,000	< 0.01
Lactate clearance (high/low)	5.3	1.67–16.8	0.01	7.10	1.71–29.5	< 0.01

**Table 5:** Net reclassification improvement and integrated reclassification improvement

	AUC	*p* value	NRI	*p* value	IDI	*p* value
Established risk factors	0.76		reference		reference	
+ Lactate clearance	0.82	0.23	0.64	< 0.01	0.121	< 0.01

Established risk factors were consisted age, sex, initial rhythm, in-/out-hospital CPA, pH, and CRP duration

## Discussion

The results of the study showed that LC was one of the independent predictors of ECPR for in-hospital mortality. Addition of LC to the established risk factors such as age, initial rhythm, in-/out-hospital CPA, pH, and CRP duration time improved NRI and IDI. Because LC is easy to calculate, is reliable, and has small fluctuations in the clinical settings, our findings might be of clinical significance.

In the case of cardiac arrest, ECPR is one of the most powerful intensive and effective treatments, as shown by previous studies [11, 12]. However, AHA guidelines limited its indication because of poor evidence [3]. In the study, we recognized that ECPR was a very useful and effective treatment for patients who after cardiac arrest.

Because ECPR and intensive care require higher cost, need more time, and are more labor-intensive, the cost/benefit should be considered [13]. Furthermore, ceasing ECPR may sometimes be recommended because of the patient’s poor prognosis. Hence, a prognostic predictor can help decide whether to continue ECPR. Although well-known predictors which have been shown in the previous reports are factors already determined before return of spontaneous circulation (ROSC) [14, 15], LC is a unique predictor because it can be calculated 6 h after starting ECPR, not before ROSC. This time lag gives us a chance to reconsider the continuation of ECPR.

In the clinical setting, patients with a lower lactate level might have a better outcome in both PCAS and sepsis. We hypothesize that the high serum lactate level with cytokinetic storm in PCAS is due to reperfusion injury, whereas that in sepsis is due to the cytokinetic storm caused by the systemic infection. In view of the cytokinetic phenomenon, sepsis is similar to PCAS, and this hypothesis is shown in a prior study [10]. Meanwhile, a previous study showed that serum lactate level is a better indicator of early goal-directed therapy than SvO_2_ [16]. Considering the similarity between sepsis and PCAS, a therapy strategy based on the serum lactate level will be more effective, even in PCAS.

A previous study showed that early goal-directed therapy is an effective strategy in sepsis [17]. Another study showed that LC is a more important indicator than SvO_2_ in sepsis [6]. We believe that early goal-directed therapy based on lactate will be an important strategy in ECPR. Our results showed that a lower serum lactate level at 6 h than the primary lactate level could be used as one of the prognostic indicators. Thus, we should consider that the prognosis after ECPR can be improved by lowering the lactate level using catecholamines, intra-aortic balloon pumping, optimization of percutaneous cardiopulmonary support, infusions, transfusions, and so forth, that is, if serum lactate-guided early goal-directed therapy will improve the prognosis of CPA and/or ECPR cost/benefit.

PCAS includes brain/myocardial disorders and systemic reperfusion injury. ECPR consists of a therapy for PCAS and treatment of the original disease caused the CPA. Therefore, with better LC, treatment of the original disease, care for the myocardial disorder after CPA, improvement of systemic circulation, and coping with systemic reperfusion injury like sepsis may be successful. Meanwhile, in the case of worse LC, failure in one or more of the abovementioned items may occur.

### Limitations

Some limitations should be considered. First, the small number of enrolled patients was not enough to determine a new evidence. Second, there were strong biases due to our ECPR exclusion criteria, e.g., age > 75 years, end-stage cancer, and strong frailty. Third, medical treatments might have also affected the results; however, we could not evaluate such data, e.g., different treatment methods for each doctor. Further studies are required to address these limitations.

## Conclusion

In our study, LC determined 6 h after ECPR significantly predict survival discharge in patients treated with ECPR after cardiac arrest. Using LC during ECPR might provide useful information whether continuing ECPR is adequate or not.

## Abbreviations

AHA: American Heart Association
CPA: Cardiopulmonary arrest
CPR: Cardiopulmonary resuscitation
ECPR: Extracorporeal cardiopulmonary resuscitation
IDI: Integrated discrimination improvement
LC: Lactate clearance
NRI: Net reclassification improvement
PCAS: Post-cardiac arrest syndrome
