# Correction to: The lactate clearance calculated using serum lactate level 6 h after is an important prognostic predictor after extracorporeal cardiopulmonary resuscitation: a single-center retrospective observational study

**DOI:** 10.1186/s40560-019-0370-8

**Published:** 2019-03-21

**Authors:** Takashi Mizutani, Norio Umemoto, Toshio Taniguchi, Hideki Ishii, Yuri Hiramatsu, Koji Arata, Horagaito Takuya, Sho Inoue, Tsuyoshi Sugiura, Toru Asai, Michiharu Yamada, Toyoaki Murohara, Kiyokazu Shimizu

**Affiliations:** 1 Cardiovascular Center, Ichinomiya Municipal Hospital, Ichinomiya, Japan; 2 Department of Emergency, Ichinomiya Municipal Hospital, Ichinomiya, Japan; 3 Department of Cardiology, Nagoya University Graduate School of Medicine, Nagoya, Japan; 4 Department of Medical Engineering, Ichinomiya Municipal Hospital, Ichinomiya, Japan; 5 Department of Cardiology, Ichinomiya Municipal Hospital, 2-2-22 Bunkyo, Ichinomiya City, Aichi 491-8558 Japan

**Correction to: J Intensive Care**

**https://doi.org/10.1186/s40560-018-0302-z**

In the original publication of this article [1], the values of PH in the Table 4 are wrong. Other values remain unchanged. The numbers are revised in the updated Table below:

**Table 4:** Multi-logistic Analysis for Surviving Discharge

	Univariable			Multivariable		
	Odds ratio	Confidential interval	*p* value	Odds ratio	Confidential interval	*p* value
age	0.98	0.95–1.03	0.63	1.02	0.96–1.10	0.49
Initial rhythm (VF and pulseless VT/ Asystole and PEA)	1.47	0.51–4.22	0.46	1.42	0.32–6.27	0.64
Location(out/in)	1.17	0.42–3.26	0.64	10.0	1.35–75.0	0.01
CPR duration	1.02	0.99–1.05	0.22	1.08	1.01–1.12	0.02
pH	2.52	1.24–5.09	0.03	7.24	4.16–15.0	< 0.01
Lactate clearance (high/low)	5.3	1.67–16.8	0.01	7.10	1.71–29.5	< 0.01
